# Effects of different transcranial magnetic stimulations on neuropathic pain after spinal cord injury

**DOI:** 10.3389/fneur.2023.1141973

**Published:** 2023-07-13

**Authors:** Chuanmei Yang, Yunfeng Bi, Luoman Hu, Lili Gong, Zhanfei Li, Nanyang Zhang, Qiang Wang, Jiang Li

**Affiliations:** ^1^Department of Rehabilitation Medicine, The Affiliated Hospital of Qingdao University, Qingdao, China; ^2^Medical Research Center, The Affiliated Hospital of Qingdao University, Qingdao, China

**Keywords:** repetitive transcranial magnetic stimulation (rTMS), intermittent theta burst stimulation (iTBS), neuropathic pain, spinal cord injury, non-invasive stimulation

## Abstract

**Introduction:**

Repetitive transcranial magnetic stimulation (rTMS) is an effective non-invasive cortical stimulation technique in the treatment of neuropathic pain. As a new rTMS technique, intermittent theta burst stimulation (iTBS) is also effective at relieving pain. We aimed to establish the pain-relieving effectiveness of different modalities on neuropathic pain. The study was conducted in individuals with spinal cord injury (SCI) and different modalities of rTMS.

**Methods:**

Thirty-seven individuals with SCI were randomly allocated to three groups, in which the “iTBS” group received iTBS, the “rTMS” group received 10 Hz rTMS, and the “iTBS + rTMS” group received iTBS and 10 Hz rTMS successively of the primary motor cortex 5 days a week for 4 weeks, and they all underwent the full procedures. The primary outcome measure was change in the visual analog scale (VAS), and the secondary outcomes were measured using the Hamilton Rating Scale for Depression (HAM-D) and the Pittsburgh Sleep Quality Index (PSQI). All the outcomes were evaluated at 1 day before stimulation (baseline), 1 day after the first week of stimulation (S1), and 1 day after the last stimulation (S2).

**Results:**

The VAS scores showed significant pain improvement after 4 weeks of stimulation (*p* = 0.0396, *p* = 0.0396, and *p* = 0.0309, respectively) but not after 1 week of stimulation. HAM-D scores declined, but the decreases were not significant until 4 weeks later (*p* = 0.0444, *p* = 0.0315, and *p* = 0.0447, respectively). PSQI scores were also significantly decreased after 4 weeks of stimulation (*p* = 0.0446, *p* = 0.0244, and *p* = 0.0088, respectively). Comparing the three modalities, VAS, HAM-D, and PSQI scores at S1 showed no differences, and, at S2, VAS scores showed significant differences (*p* = 0.0120; multiple comparisons showed significant differences between iTBS and iTBS + rTMS, *p* = 0.0091), while the HAM-D and PSQI scores showed no differences.

**Discussion:**

The primary and secondary outcomes all showed significant improvement, indicating that the three different modalities were all effective at relieving the pain. However, not all the three stimulations were of same effectiveness after treatment; there were statistical differences in the treatment of neuropathic pain between iTBS as a priming stimulus and as a single procedure.

## Introduction

Spinal cord injury (SCI) frequently leads to neuropathic pain. It is reported that 81% of individuals with SCI experience chronic pain and 86% of individuals with pain have neuropathic pain, which has a significant impact on daily living and quality of life (1). Pharmacological and non-pharmacologic treatments have been used to reduce intractable pain, but these approaches frequently do not achieve adequate pain control in many cases (2, 3). Pharmacotherapies are associated with adverse drug reactions, including dizziness, somnolence, and nausea. Some invasive techniques require surgical procedures or trial periods, which significantly lower compliance, sometimes leading to discontinuation before a therapeutic effect can be achieved (4, 5). Therefore, there is an urgent need for better treatment options.

Non-invasive cortical stimulation techniques have been used to control chronic neuropathic pain by either inhibition or interruption of thalamic pain signals and other hyperactive localization pain networks (6). Repetitive transcranial magnetic stimulation (rTMS) as a non-invasive, promising cortical stimulation technique has been suggested to be more effective in the treatment of neuropathic pain of central origin (7, 8). Therefore, there is substantial evidence showed effective pain management when applying rTMS to the primary motor cortex (M1) (5, 9, 10). There are several parameters associated with the effectiveness of rTMS like stimulation site, field orientation, frequency, intensity, and duration of stimulation (11). A newer form of rTMS, known as intermittent theta burst stimulation (iTBS), displays faster, more robust action compared to conventional protocols, with excellent tolerability, provided that safety recommendations are followed (12, 13). iTBS delivers 600 pulses in just 3 min 20 s, yet shows similar or more potent excitatory effects than conventional 10 Hz rTMS (14). iTBS has been approved by the U.S. Food and Drug Administration (FDA) for treatment-resistant depression, revealing its potential in neuromodulation therapy. iTBS recently been introduced in neuropathic pain in SCI, even though these parameters have not yet been optimized. It has also been reported that the analgesic effects of 10 Hz rTMS delivered to M1 can be enhanced by iTBS priming (15, 16). Nevertheless, we still do not know whether 3 min iTBS sessions are superior to 10 Hz sessions or iTBS-primed 10 Hz sessions.

Therefore, we conducted a randomized controlled trial to compare the three modalities on neuropathic pain in SCI individuals, aiming to establish the pain-relieving effectiveness of different TMS modalities. We hypothesized that all three modalities of rTMS would achieve comparable analgesic effects.

## Methods

### Individuals and randomization

Thirty-nine individuals with SCI were recruited from the Department of Rehabilitation Medicine between August 2020 and June 2021 to participate in the research. The inclusion criteria for the study were: (i) SCI confirmed by CT or MRI and meeting the criteria of neuropathic pain by the International Association for the Study of Pain; (ii) individuals experienced pain, depression, and sleep disorders; and (iii) the pain was not attributable to any other cause, such as rheumatologic disorders or diabetes.

The exclusion criteria included epilepsy, drug-addiction, migraine, and intracranial ferromagnetic material or implanted stimulators. All individuals provided written informed consent, and the study was approved by the Ethics Committee of the Affiliated Hospital of Qingdao University (QYFY WZLL 27661) and has been registered in the Chinese Clinical Trial Registry (ChiCTR2300072864).

**Figure 1:**
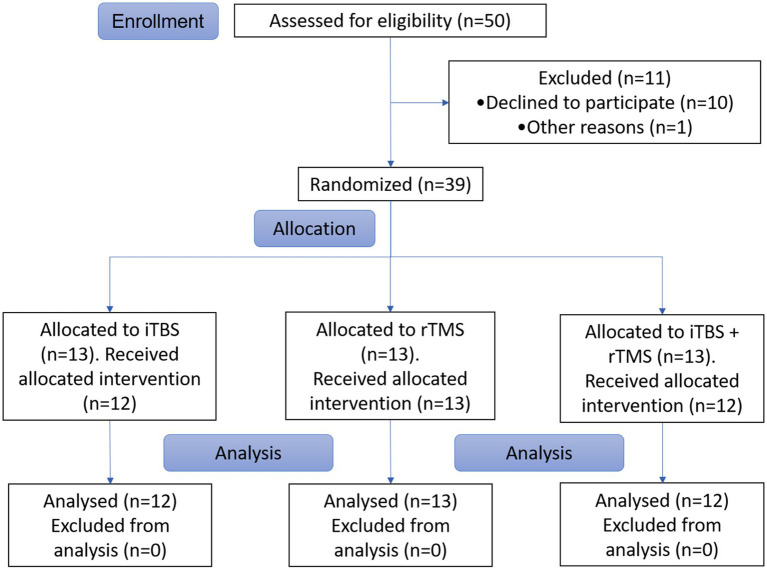
Flowchart of the study.

This study was designed as a prospective, randomized clinical trial. A computer-generated randomization schedule was used to separate the individuals into three groups, with one group named “iTBS” receiving iTBS, another named “rTMS” receiving 10 Hz rTMS, and the other named “iTBS + rTMS” receiving iTBS and 10 Hz rTMS successively (Figure 1).

### Procedures

The individuals received the stimulations while sitting in a comfortable chair or their wheelchair in the TMS Treatment Room, Department of Rehabilitation Medicine.

In the iTBS sessions, iTBS was delivered in a stimulation pattern of triplet 50 Hz bursts, repeated at 5 Hz, 2 s on and 8 s off, with 600 pulses per session for a total duration of 3 min 20 s. The stimulation was performed in each session per day at an intensity equal to 80% of the resting motor threshold (rMT) (17). A circular coil connected to a YRD CCY-I Magnetic Stimulator (Yiruide, Wuhan, China) was used for stimulation of the contralateral motor cortex (M1). Briefly, the coil was fixed on the three-dimensional adjustable arm, and a circular coil with a diameter of 12.5 cm was placed in the M1 region corresponding to the surface of the individual’s skull, and stimulation was given.

In the rTMS sessions, 30 trains of stimulation with a frequency of 10 Hz were applied at the same site and intensity (80% rMT). The duration of each train was 5 s and the interval between two trains was 25 s. In total, 1,500 pulses in 15 min of stimulation were performed in each session per day.

In the priming stimulation sessions, stimulation was administered starting with intermittent theta burst (iTBS) delivered at 80% rMT and followed 2 min later by 10 Hz rTMS delivered at 80% rMT for a total of 2,100 pulses.

The initial treatment comprised 20 sessions in total, which consisted of oncedaily sessions (on weekdays, i.e., five sessions a week).

### Clinical assessment

Pain was assessed by an investigator blinded to the type of stimulation individuals were receiving, using the visual analog scale (VAS).

Living with chronic pain is linked with depression for many people. Research shows that depression is more prevalent in people that live with chronic pain than those who live with other illnesses (18). The multiple-item Hamilton Rating Scale for Depression (HAM-D) questionnaire was designed to measure the frequency and intensity of depressive symptoms in individuals, and is considered the gold standard for assessing the severity of depression and is widely used in research (19). We used HAM-D (30 mild: score 8–13; moderate: 14–18; and severe: 419) to assess the depression of individuals induced by pain. Apart from the pain itself, some people with chronic pain also experience one or more sleep disorders (20), such as obstructive sleep apnea or restless legs syndrome. One in four people with chronic pain also have a sleep disorder. The Pittsburgh Sleep Quality Index (PSQI) is an effective instrument used to measure the quality and patterns of sleep in adults (21). We used the PSQI to evaluate the individuals’ sleep quality.

**Figure 2:**
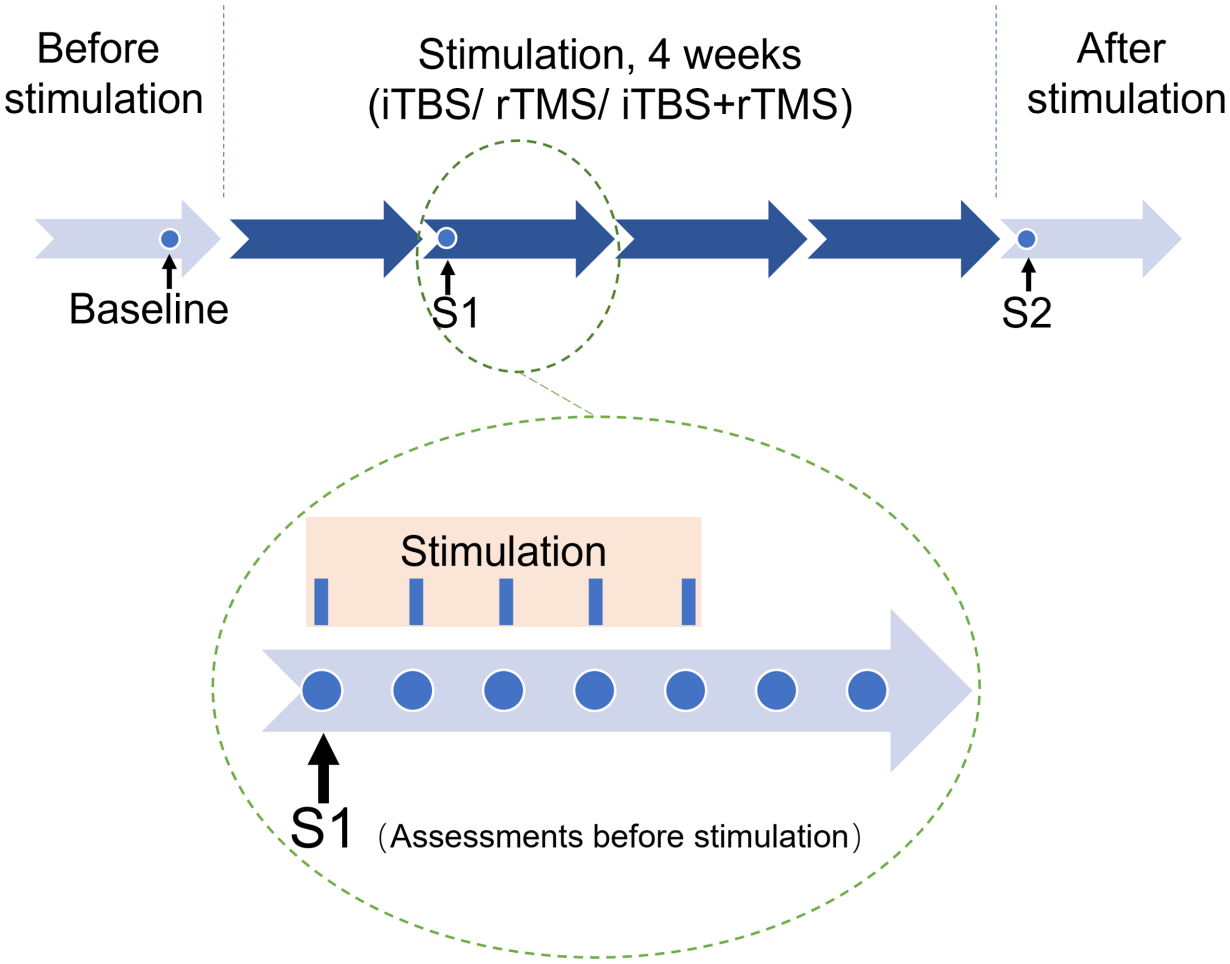
Stimulation protocol and clinical evaluation.

Pain and mood assessments were performed at baseline, 1 day after the first week of stimulation (S1), and 1 day after the last stimulation (S2). The analgesic effects were assessed as the primary outcome, and the effects on mood symptoms and sleep quality were assessed as secondary clinical outcomes (Figure 2).

### Statistics

For intragroup comparisons across all time-points, we used repeated-measures analysis of variance (ANOVA) with Bonferroni correction. At the baseline assessments prior to the stimulation intervention, the mean values of all the assessments were compared between the groups, using one-way ANOVA or the Mann–Whitney test for independent samples for continuous data or the chi-squared test for categorical data. The time-points at baseline, S1, and S2 were employed as the within-individual factors, and the groups iTBS, rTMS, and iTBS + rTMS were used as the between-individual factors. Data analysis was performed using GraphPad Prism software (v7). A *p* value less than 0.05 was considered statistically significant.

## Results

Thirty-seven individuals with SCI were recruited for this study, and all the individuals tolerated the study well. One individual in the iTBS group and one individual in the iTBS + rTMS group withdrew from the study for personal reasons. The characteristics of all individuals who completed the study are shown in Table 1. Table 2 shows the results of the functional outcome scores at the different time points after different stimulation application.

**Table 1:** Individuals’ characteristics.

	iTBS group (*n* = 12)	rTMS group (*n* = 13)	iTBS + rTMS group (*n =* 12)
Age (years)	49.75 ± 13.86	51 ± 16.35	46.94 ± 12.95
Sex (male/female)	9/3	7/6	7/5
Mean time after SCI (months)	14.77 ± 2.15	15.63 ± 2.41	16.45 ± 3.01
Duration of pain (months)	13.58 ± 1.46	14.35 ± 3.16	15.21 ± 2.33
ASIA (A/B/C/D)	0/4/6/2	0/6/4/3	0/7/4/1

iTBS, intermittent theta burst stimulation; rTMS, repetitive transcranial magnetic stimulation; SCI, spinal cord injury; ASIA, American Spinal Cord Injury Association Impairment Scale.

**Table 2:** Primary and secondary functional outcome scores of the individuals in the three groups.

	Baseline	S1	S2
VAS			
iTBS	7.03 ± 0.78	6.37 ± 0.73	4.85 ± 0.62
rTMS	7.27 ± 1.19	6.34 ± 1.06	4.38 ± 0.59
iTBS + rTMS	7.34 ± 1.32	6.07 ± 0.54	4.06 ± 0.63
HAM-D			
iTBS	16.51 ± 2.79	14.59 ± 1.37	10.1 ± 1.12
rTMS	17.89 ± 3.26	15.24 ± 2.79	10.08 ± 1.03
iTBS + rTMS	18.72 ± 3.86	15.91 ± 3.15	10.25 ± 0.96
PSQI			
iTBS	14.74 ± 2.19	12.37 ± 2.23	8.86 ± 1.68
rTMS	15.36 ± 2.36	12.45 ± 2.71	8.59 ± 1.54
iTBS + rTMS	15.81 ± 2.25	12.51 ± 2.12	8.07 ± 1.48

### Evaluation of the primary outcome of the individuals in the three groups

**Figure 3:**
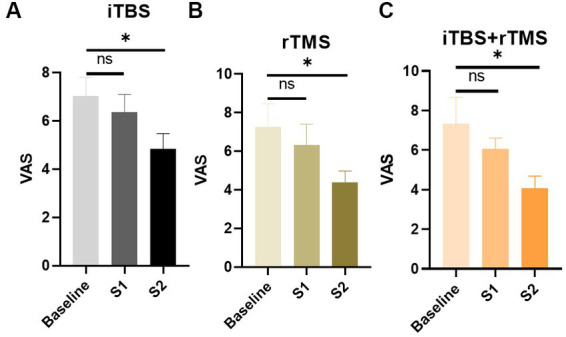
Evaluation of the pain of the individuals by VAS scores in the three groups. **(A)** VAS scores of the individuals in the iTBS group. **(B)** VAS scores of the individuals in the rTMS group. **(C)** VAS scores of the individuals in the rTMS + iTBS group. The short line above the bar chart represents the comparison between the baseline and S1; the long line above the bar chart represents the comparison between the baseline and S2. Data are mean ± s.e.m. ^*^p < 0.05; ns, not significant.

In the iTBS group, assessed at three time points, VAS scores showed a downward trend. Compared with the baseline, the decrease of VAS scores at S1 was not significant (*p* = 0.5431), while at S2 they were significant (*p* = 0.0396, Figure 3). The results demonstrated that iTBS was effective at relieving the pain by the fourth week of stimulation.

Similarly, in the rTMS and iTBS+ rTMS groups, VAS scores showed a significant decrease at S2 (*p* = 0.00396, and *p* = 0.0309, respectively), but not at S1 (*p* = 0.5650, and *p* = 0.3688, respectively), indicating that rTMS or rTMS combined with iTBS could also effectively lower the level of pain.

### Evaluation of the secondary outcome of the individuals in the three groups

**Figure 4:**
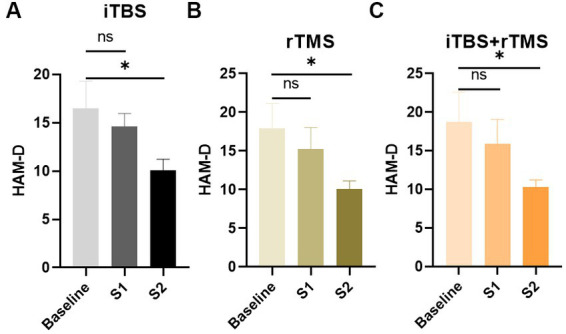
Evaluation of the depression of the individuals by HAM-D scores in the three groups. **(A)** HAM-D scores of the individuals in the iTBS group. **(B)** HAM-D scores of the individuals in the rTMS group. **(C)** HAM-D scores of the individuals in the rTMS + iTBS group. The short line above the bar chart represents the comparison between the baseline and S1; the long line above the bar chart represents the comparison between the baseline and S2. Data are mean ± s.e.m. ^*^p < 0.05; ns, not significant.

Figure 4 shows that the HAM-D scores in the iTBS, rTMS, and iTBS + rTMS groups all decreased during the week preceding stimulations, while there were no significant differences between the baseline and S1 (*p* = 0.5431, *p* = 0.5427, and *p* = 0.5785, respectively). When comparing the HAM-D scores at baseline versus S2, the differences were significant (*p* = 0.0444, *p* = 0.0315, and *p* = 0.0447, respectively); the individuals who received the three modes of stimulation all showed significant effects of alleviating depression.

**Figure 5:**
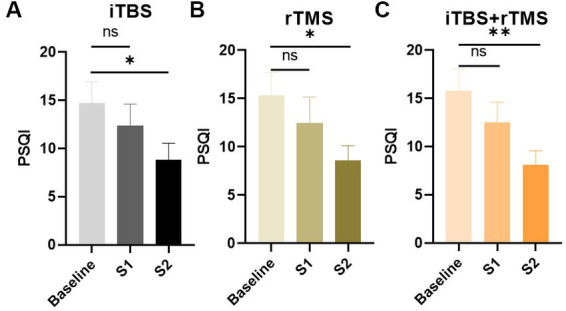
Evaluation of the sleep quality of the individuals by PAQI scores in the three groups. **(A)** PAQI scores of the individuals in the iTBS group. **(B)** PAQI scores of the individuals in the rTMS group. **(C)** PAQI scores of the individuals in the rTMS + iTBS group. The short line above the bar chart represents the comparison between the baseline and S1; the long line above the bar chart represents the comparison between the baseline and S2. Data are mean ± s.e.m. ^*^p < 0.05, ^**^p < 0.01; ns, not significant.

Figure 5 illustrates the results for the PSQI scores. Similar to the pain scores and depression scores, after 1 week treatment, sleep quality was improved, but there were no significant differences (*p* = 0.4563, *p* = 0.4260, and *p* = 0.2973, respectively). However, after the 4 weeks treatment, sleep quality was significantly improved (*p* = 0.0446, *p* = 0.0244, and *p* = 0.0088, respectively).

### Comparison of outcomes among the three groups at different timepoints

**Figure 6:**
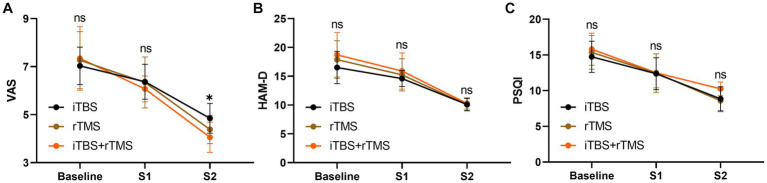
Intergroup comparison of outcomes at different timepoints. **(A)** VAS scores of the individuals at three timepoints among the three groups. **(B)** HAM-D scores of the individuals at three timepoints among the three groups. **(C)** PSQI scores of the individuals at three timepoints among the three groups. Data are mean ± s.e.m. ^*^p < 0.05; ns, not significant.

Before treatment, the VAS, HAM-D, and PSQI scores in the three groups were not statistically different (*p* = 0.7784, *p* = 0.2728, and *p* = 0.5172, respectively), meaning they were comparable in the indicators among the three groups (Figure 6). After treatment, there were no significant differences in VAS, HAM-D, and PSQI scores at S1 among the three groups in order (*p* = 0.6118, *p* = 0.4592, and *p* = 0.9896, respectively). At S2, VAS scores were significantly different (*p* = 0.0120), while HAM-D and PSQI scores were not significantly different among the three groups (*p* = 0.9921, and *p* = 0.6850, respectively). Accordingly, we conducted multiple comparisons of VAS scores at S2, and the result showed that there was significant difference between the iTBS group and the iTBS+ rTMS group (*p* = 0.0091).

## Discussion

Neuropathic pain caused by SCI is considered a purely pathological, maladaptive consequence of central nervous system damage and inflammation rather than a potentially useful protective response. TMS has advantages over drugs and invasive electrical stimulation in treating it (22). TMS can stimulate neurons at various horizontal levels, inducing not only biological effects but also influencing local and functionally related cortical functions from a distance to achieve regional reconstruction of cortical functions. This leads to an increased number of nerve impulses traveling down the spinal cord to motor neurons, maximizing residual nerve fiber connections. As a result, this not only contributes to nerve regeneration after SCI but also enhances brain plasticity.

In the current clinical study, we analyzed the effects of iTBS, rTMS, and the combination of the two stimulations on neuropathic pain in individuals with SCI. There is much research proposing the use of rTMS on the M1 for the treatment of neuropathic pain (23, 24). Previous studies have shown that rTMS of M1 can reduce the average pain and the most severe pain after SCI (25, 26). Our study was consistent with them. There are reports that have suggested 20 Hz as a suitable frequency content for rTMS, while in our clinical practice, most of the individuals we treated could not endure that frequency content, and 10Hz was more suitable.

The relief effect of iTBS on neuropathic pain after SCI is not well studied, though physiological properties and comfort of application make iTBS a theoretical alternative to conventional rTMS. Kohutova et al. (27) reported that a single-session of iTBS could relieve facial pain in 10 individuals after 60 min after treatment, which could last 2 weeks. Recently, Kim et al. (28) reported that with iTBS treatment, a group of 15 individuals gained central pain relief. In the present study, we assessed the effectiveness of iTBS on neuropathic pain after SCI. The results showed that after 4 weeks treatment, iTBS was indeed able to reduce pain compared to the baseline.

In our study, after 4 weeks of stimulation, the VAS scores showed significant differences among the three groups, indicating that iTBS priming partly produced greater analgesia than the other protocols. It requires sufficient stimulation time for better pain relief based on our result, because after 1 week stimulation, the VAS scores had no difference. iTBS induces greater and longer-lasting changes in cortical excitability compared to conventional rTMS at 10 Hz, which is known to potentiate cortical excitability in normal individuals (14). It is not clear whether the superiority of iTBS over high frequency (HF) rTMS in terms of cortical excitability can be reliably applied to a heterogeneous population of individuals with neuropathic pain. Mechanically, motor stimulation on distant pain-related areas could modulate pain. It may be a better fit between HF-rTMS and sensorimotor networks that explains its superiority to iTBS; synaptic efficacy and plasticity may be increased when motor cortex oscillations are synchronized with rTMS, thus enhancing the functional connectivity of the motor cortex with distant structures involved in pain regulation.

Our results demonstrated that three different modalities of TMS were all effective at relieving pain. However, not all three stimulations were of same effectiveness after treatment; there were statistical differences in the treatment of neuropathic pain between iTBS as a priming stimulus and as a single procedure. Lefaucheur et al. (15) found that the analgesic effects of “conventional” 10 Hz rTMS delivered to M1 can be enhanced by TBS priming, while our study did not support that. Interestingly, our results showed that iTBS as a priming stimulus was more effective than as a single procedure. rTMS therapy often requires daily treatments for over a month, which can be inconvenient for patients who need to take time off work or find transportation. Furthermore, it is worth noting that iTBS sessions have been found to be completed in approximately 30 min less time than 10 Hz rTMS. This means that patients can receive the same level of treatment effectiveness without having to spend as much time undergoing therapy. Moreover, our study has suggested that there are no significant differences in prognostication of treatment response between these two modalities. This could also have important implications for clinicians who are looking to optimize their treatment protocols and reduce the burden on patients. By increasing their utilization of iTBS over 10 Hz rTMS, they may be able to provide more efficient care while still achieving positive outcomes. Of course, it is important to note that every patient’s needs and circumstances are unique, and what works best for one person may not work as well for another. However, this study provides valuable insights into the potential benefits of using iTBS as a preferred modality for certain types of patients or conditions.

Overall, our research underscores the importance of continuing to explore new approaches and techniques in order to improve patient outcomes and enhance our understanding of how different treatments can impact mental health and wellbeing. The conclusion we reached is limited due to the small sample size. Individuals with SCI are relatively rare, so type II errors may be responsible for the results of the study that did not demonstrate the significant differences of the three modes of stimulation. Future studies in larger population are needed to reduce this risk. Moreover, we did not perform a follow-up. We measured all the variables as soon as the treated sessions were finished. To some extent, positive results are to be expected, but how long the pain relief effect could last after the treatment is unknown, which differs with some other studies. Nevertheless, middle-term pain relief provided by different modes of stimulation are encouraging and suggests the need for future studies with a larger sample size which may reveal clinically relevant differences.

## Data availability statement

The original contributions presented in the study are included in the article/supplementary material, further inquiries can be directed to the corresponding authors.

## Ethics statement

The studies involving human participants were reviewed and approved by The Ethics Committee of the Affiliated Hospital of Qingdao University. The patients/participants provided their written informed consent to participate in this study.

## Author contributions

JL, QW, and CY contributed to the conception and design of the study. CY and YB organized the database. CY, LH, and LG performed the statistical analysis. CY wrote the first draft of the manuscript. ZL and NZ wrote sections of the manuscript. All authors contributed to the article and approved the submitted version.

## Conflict of interest

The authors declare that the research was conducted in the absence of any commercial or financial relationships that could be construed as a potential conflict of interest.

## Publisher’s note

All claims expressed in this article are solely those of the authors and do not necessarily represent those of their affiliated organizations, or those of the publisher, the editors and the reviewers. Any product that may be evaluated in this article, or claim that may be made by its manufacturer, is not guaranteed or endorsed by the publisher.
